# The perceptions and perspectives of patients and health care providers on chronic diseases management in rural South Africa: a qualitative study

**DOI:** 10.1186/s12913-015-0812-5

**Published:** 2015-04-08

**Authors:** Eric Maimela, Jean-Pierre Van Geertruyden, Marianne Alberts, Sewela EP Modjadji, Herman Meulemans, Jesicca Fraeyman, Hilde Bastiaens

**Affiliations:** Department of Medical Sciences, Public Health and Health Promotion, University of Limpopo, Turfloop campus, Private Bag X1106, Sovenga, 0727 South Africa; International Health Unit, University of Antwerp, Universiteitsplein 1, 2610 Wilrijk, Belgium; Department of Sociology and Research Centre for Longitudinal and Life Course Studies, University of Antwerp, Sint-Jacobstraat 2, BE-2000 Antwerpen, Belgium; Research group Medical Sociology and Health Policy, University of Antwerp, Universiteitsplein 1, 2600 Wilrijk, Belgium; Department of Primary and Interdisciplinary care, University of Antwerp, Universiteitsplein 1, 2610 Wilrijk, Belgium; Centre for Health Systems Research & Development, University of the Free State, Bloemfontein, South Africa

**Keywords:** Chronic disease management, Chronic patients, Knowledge, Medication supply, Trainings, Nurses, Qualitative research

## Abstract

**Background:**

Preventive health care represents the future for health care delivery in South Africa to improve management of chronic diseases as this has been implemented for some time in several countries to tackle the increasing burden of chronic diseases. Individual person’s health is unique, as they move in and out of chronic and acute health care phases, there is need to integrate chronic and acute care constructs to improve continuity of care and maximize health and improve wellbeing. The aim of this study was to determine the perceptions and perspectives of chronic patients’ and nurses regarding chronic disease management in terms of barriers, facilitators and their experiences.

**Methods:**

To meet our aim we used qualitative methods involving the collection of information by means of focus group discussions in Dikgale Health and Demographic Surveillance System (HDSS). All data was recorded, transcribed verbatim and analysed using data-driven thematic analysis.

**Results:**

Our study showed that chronic disease patients have a first contact with health care professionals at the primary health care level in the study area. The main barriers mentioned by both the health care workers and chronic disease patients are lack of knowledge on chronic diseases, shortage of medication and shortage of nurses in the clinics which causes patients to wait for a long periods in a clinic. Health care workers are poorly trained on the management of chronic diseases. Lack of supervision by the district and provincial health managers together with poor dissemination of guidelines has been found to be a contributing factor to lack of knowledge in nurses among the clinics within the study area. Both patients and nurses mentioned the need to involve community health workers and traditional healers and integrate their services in order to early detect and manage chronic diseases in the community.

**Conclusions:**

Nurses and chronic disease patients mentioned similar barriers to chronic disease management. Concerted action is needed to strengthen the delivery of medications at the clinics, improve the chronic disease knowledge for both nurses and patients by conducting in-service trainings or workshops, increase the involvement of community health workers and establish a link (through formal referral system) with traditional healers.

## Background

The global health care system is troubled by the increasing chronic disease burden [1,2]. Chronic disease management (CDM) helps people to keep as healthy as possible through the prevention and early detection of complications, and management of these chronic diseases. CDM is a public health care approach emphasizing and encouraging individuals living with chronic diseases to maintain their independence by managing their health conditions and therefore maintain functional capacity [3]. This is important for the concerned public health officials as maintaining a healthy quality of life on individual level also creates stability and equality among society members as a whole. With the growing elderly population in South Africa and changing life styles, chronic diseases have become a primary factor provoking a rise in medical costs and decreased quality of life [4].

Organizational or structural interventions are crucial for chronic disease management as the management of chronic diseases is distinct from health care for acute problems [5]. Thus, there is a need to refocus and strengthen the primary health care as chronic diseases need opportunistic case finding for assessment of risk factors, early detection of disease and identification of high risk status [5]. Successful management of chronic conditions requires behavioural and lifestyle adjustments to minimize functional limitations and disability [6]. As chronic patients are in many ways their own primary carers, their needs and preferences must be taken into account in the development of management plans [5]. This may be accomplished by organizational interventions guided by an appropriate theoretical framework fitting the health problem of interest to change behaviour [7].

The encounters between health care professionals and chronically ill patients may be variously sporadic or on-going, occasional or intensive, and may involve one primary care provider or an array of specialist practitioners. This variability results in a threat to continuity of care which is essential in chronic care [8,9]. The vast majority of CDM is typically conducted by the patient in his or her personal environment. Therefore, encounters between patients and their health care professionals become a critical intersection for information exchange, decision-making and motivation [9]. The ability of the health care professional to engage in effective communication may consequently make a profound difference in whether the encounter supports or discourages decisions and subsequent actions that will optimize the patient’s ability to live as well as possible with that particular disease [9].

Limited data about patients’ perspectives of active participation suggest variability in the extent to which patients wish to participate, barriers to their participation and the need for physicians to adopt new paradigms with respect to patient participation [10]. Patient empowerment, patient involvement and shared decision making are frequently used concepts as patients are increasingly encouraged to take up an active role in knowing and managing their own health, by expressing their concerns, preferences and participating in medical decisions [11]. It is believed that informed patients improve their decisions by collaborating with their health-care providers [12]. This results in increased patient’s involvement leading to a positive effect on the health outcomes [11]. Increasing patient involvement in health care innovation has become a national priority and yet in practice, most interventions are still designed without the input of the patients they are intended to benefit. This gap between principle and practice may be due to a lack of knowledge and creates difficulties to operationalize the collaboration between health system leaders, researchers and patients [13]. Thus, the research question for this study was two-fold. First, how do individuals with distinct chronic diseases experience their encounters with professional health care providers (HCP) and what are their expectations and suggestions? And secondly, how do HCP perceive the current CDM and what are their expectations and suggestions for the future CDM? Hence, the objective of our study was to describe chronic patients’ and HCPs (nurses) perspectives on CDM in a rural community Limpopo Province, South Africa.

## Methods

Qualitative research design was used aiming to develop a comprehensive understanding of how patients and nurses perceive and experience the current organisation and daily practice of the care for chronic conditions in a rural area in South Africa. This was done to research the reality of chronic disease management from the perspective of the nurses and patients in the rural area of Dikgale. In this approach the past experience of the participants (nurses and patients) was respected, construction of knowledge was interactive, inductive, and collaborative, and questions were valued [14]. A constructivist approach allows themes of importance to emerge as they are constructed by participants.

Ethical approval to conduct the study was sought from the University of Limpopo Medunsa Research Committee which is a research ethics committee for University of Limpopo (MREC/HS/05/2013:PG). An institutional or departmental approval to conduct the study in health facilities was granted by the Provincial Research Committee which serves as the review board for the Department of Health in Limpopo Province. Written informed consent for participation was obtained from the participants and they were provided with information leaflet about the study.

### Research setting and sampling

Health care service in Dikgale HDSS is provided by three clinics. Organisation of health services for chronic diseases in the study area are based on primary care which has the central role as a coordinating hub, but it is not complemented by more specialized and intensive care settings, such as diagnostic labs, specialty care clinics, hospitals and rehabilitation centres. Within the study area, community health workers and traditional healers also provide health services.

Purposive sampling method was used as this involves selection of informants based on an important characteristic under study such as chronic patients diagnosed with diabetes mellitus and hypertension, as these are the most prevalent chronic diseases in the study area and are, in principle handled at the clinics. The nurses had experience with CDM in the region and were working in the clinics within the study area. We selected informants with the assistance of clinic nurses and clinic manager after we defined participants for our focus groups [15-17]. The primary data for this study were drawn from audiotape-recorded interviews with six focus group discussions (FGDs) (three for chronic patients and three for HCPs). Approximately 8 to 12 patients diagnosed with either diabetes mellitus or hypertension were selected to form a FGD per participating clinic. Gender balance in selection of respondents was adhered to where possible but majority of the participants were females (most nurses in these rural clinics are females and for patients most men were not available during the conduct of the study. This might be due to the fact that more males were working). Dikgale HDSS has a large proportion of population working as migrant workers, either on long- or short-term bases. These migrant workers work in nearby town as labourers in commercial agriculture or as domestic workers, or tourist areas, with some away on an extended basis in the mining sector [18]. Three FGDs were conducted for chronic patients in total. Due to shortage of nurses in the rural clinics in Limpopo Province, 6 to 10 nurses were selected to form a FGD per participating health facility. Thus, in total three FGDs were done for nurses. As the majority of the nurses are females, it was not possible to keep a gender balance.

### Data collection

A semi-structured interview guide was developed and pilot-tested for nurses and patients. The interview guide for nurses consisted of open-ended questions on the following topics:

- Interventions/programmes in place to manage chronic diseases
- Concerns from chronic disease patients and nurses regarding chronic disease management
- Role of health district in the provision of essential healthcare
- Community awareness of chronic diseases
- Community participation in health service delivery
- Readiness (willingness and ability/capacity) of the communities to engage in health service delivery
- Community effectiveness in the involvement on chronic disease prevention and management.

The interview guide for chronic patients consisted of open-ended questions on the following themes and each had subsequent questions:

- Beliefs on non-communicable diseases and their risk factors
- Health seeking behaviors
- Experiences with the current health care system

### Data management and analysis

Because of the exploratory nature of the study, we applied an inductive thematic analysis method [19,20]. Sentences, phrases, paragraphs or lines were linked with codes; codes were then compared across the whole data set to identify variations, similarities, patterns and relationships. Reflections and ideas were written related to sections of data to abstract from the data and deepen analysis (memo writing). Codes were grouped to create a smaller number of themes that distilled the key issues identified and relationships between themes are then identified to create an explanatory diagram. This was initially done separately for nurses and patients. In a final step, themes were grouped for the whole dataset.

The principal investigator (ME) coded all transcripts. Checks of transcripts codes and information exchange with the last authors (BH and FJ) were regularly conducted so as to ensure consistency.

## Results

The study population included front line primary health care nurses working at the three clinics and chronic disease patients who were diagnosed and treated in these clinics. The gender distribution of nurses who formed part of the FGDs was dominated by females in all the participating clinics. The experience of nurses for delivery of health related services who participated in the FGDs ranged from one year to more than ten years of service. Majority of the nurses (29%), were having work experience of between 4–6 years and 10 years and above.

Table 1 gives an overview of the main themes and subthemes for nurses and patients separately. The result section below is organised around themes summarizing both patients and nurses views: organisation of health services collaboration with traditional health practitioners, traditional authority supporting health service delivery, functioning of the clinic committees, utilisation of community health workers, needs for knowledge and training regarding chronic conditions and resources needed for CDM.

**Table 1:** **Schema as a guide for readers**

**Patients**	
Needs in relation to knowledge and education	Knowledge on causes, symptoms and treatment of chronic diseases
	Information via several channels (TV, radio, clinics, schools, traditional healers)
Needs related to health care organization	Availability of medication at clinics
	Decrease waiting time Health Care Providers (HCP) to come in time)
	Provide support to manage condition close-by in the community (role for community health workers)
Mixed perceptions on relation with health care providers and home based carers	Positive experiences with nurses and home based carers as they give information on disease and treatment
	A number of patients lack respect for home base carers since they view them as not well trained
Opportunities patients see in the community	Train and involve traditional healers/leaders and home based carers on chronic disease management
	Empower the community by information campaigns
	Involve community through functional clinic committees
**Nurses**	
Role of clinics and nurses	Disseminate health information in the community
	Organize semi-annual review of patients by doctors
	Organize weekly dedicated days for chronic conditions by nurses
Collaboration with other health care providers	There is a good collaboration with home based carers who refer patients to clinics and participate in follow up
	Minimal collaboration with traditional healers and health authority (no meetings, very few referrals from THP) in clinics
	Interdisciplinary meetings with nurses, home based care and traditional healers in one facility
Barriers for good chronic disease management	Limited availability of medication, functional equipment and transport for nurses
	Shortage of nurses and other health professions
	Lack of training for nurses and home based carers on chronic diseases
	Lack of facilities for physical activity

### Organization of health services and roles of nurses in relation to chronic disease management

Our study findings revealed patients are first seen, diagnosed and managed at the clinics before they are referred to the doctors and social workers for further management of any complications at the hospital (secondary health care level) which is situated 15 kilometres from the study area.

#### Quotations

*“(…) the system we normally use is referral system wherein we refer patients to the doctor or social worker for medical management”.*

(*FGD: Nurses*).

The nurses in the clinics contribute to the management of chronic diseases by providing patients with medications, disseminating health information during consultations and to the community. This is mainly done through health talks in the morning, door to door campaigns and home visits.

#### Quotations

*“(…) we encourage our patients to comply with treatment given to them” “(…) we give health education in the morning to all patients and treatment”.*

(*FGD: Nurses*).

There are also doctors from hospital who visit the clinics periodically to review the progress of the patients on the medications given to them.

#### Quotations

*“(…) there are doctors from the hospital who come to see our patients on a fortnight basis”. “(…) for chronic diseases we have organised patients to come to the clinic on dedicated days only when the doctor is visiting the clinic”.*

(*FGD: Nurses*).

Our study findings showed that the nurses in health facilities as they are providing basic preventive, promotive and curative care they do not get proper support supervision from managers in the province and district including the local area under its jurisdiction.

#### Quotations

*“(…) I think their* (District and Province) *role is to support the officers or clinic personnel to see if whether there is enough medications, to see that there are enough instruments or equipment’s, to see that the community is well managed in all the spheres by well-trained officers, but they are not doing all these.*

*(FGD: Nurses).*

*“(…) no support except when they come to look for problems that are caused by nurses. This is a fault finding mission” “(…) there is no monitoring and evaluation from the district health team as they only come when there are problems in the clinic more especially when there is a patient who died after visiting the clinic or died while in the clinic”*

*(FGD: Nurses).*

### Collaboration with traditional health practitioners

It was found that traditional health practitioners do not refer patients to the clinics unless the patients are showing signs of complications.

#### Quotations

*“(…) sometimes the traditional healers don’t really refer patients to us in the clinic because we don’t know them but with people who know them they will tell that that patient was brought here by the traditional healer. This mostly happens when patients are at a complicated stage. We therefore realise that traditional healers refer patients when they stuck not knowing what to do.”*

(*FGD: Nurses*).

Patients carry hope that their conditions will heal with help from traditional healers. Nurses literally states that traditional healers need to be educated as they also give patients medications and nurses do call for formal referral.

#### Quotations

*“(…) patients are then given concussions which make them to vomit and have diarrhoea, later when the patients are dehydrated the traditional healers will bring them to the clinic to get a drip and once they are rehydrated the traditional healer will tell the patients to stop taking medication which they have received from the clinic then focus on what they will give them. This is a challenge to us because patients default from treatment with the hope that they will be healed by what traditional healers are giving them. We therefore need to have a session with the traditional healers to educate them on what needs to be done for them to can properly refer patients to us at an early stage.”*

(*FGD: Nurses*).

*“(…) some of the traditional healers they don’t integrate with health services because they do their own things and some patients when they come to the clinic they reveal that they have been using the traditional medicines instead of the medication from the clinic.”*

(*FGD: Nurses).*

Our findings also showed that there is a challenge with regard to nurses working with traditional healers as they mainly do not refer patients to the clinics and there is no integration of health services.

#### Quotations

*“(…) there are no meetings held with traditional healers which is a challenge because we are unable to integrate them in our services.” “(…) lack of communication between us and the traditional healers is a challenge.” “(…) to wrap this up, there is no interaction at all between traditional healers and us in the clinic*

(*FGD: Nurses*).

### Functionality of the clinic committees

Our data showed some aspects of the organization of health services. The clinics have clinic committees in which community members represent the community and participate in the decision on health service related issues. Unfortunately, the clinic committees are not functional and this affects the provision of effective direction, meaningful support, monitoring and evaluation and strategic interventions.

#### Quotations

*“(…) our clinic committee is not functional so sometimes it is difficult to make suggestions to the nurses as we are afraid that might affect our relationship with them. “(…) it would be nice if the clinic committee is functional then we raise concerns and suggestions to them such that they can bring them to the clinic.”*

(*FGD: Chronic patients).*

*“(…) community members are not part of decision making in provision of health services because currently there is no clinic functional clinic committee.” “(…) our communities are not involved in health service delivery because the clinic committee is non-functional and their concerns sometimes are not reaching the clinic.”*

(*FGD: Nurses*).

Patients are supposed to use the suggestion box provided in the clinics to raise their concerns or challenges with regard to provision of health services in the clinics. However, most patients are not using the suggestion box.

#### Quotations

*“(…) yes it is true the suggestion box is there but we are not using it as we are not sure how will nurses react after reading our suggestions or concerns.” “(…) myself I don’t make use of the suggestion box because I’m afraid I might be victimized by nurses as there is a lot which they are not doing right. So it’s best for me to just come here and take medication then I go home without making suggestions to improve health services.”*

(*FGD: Chronic patients).*

*“(…) patients and community members are encouraged to take part in health service delivery by writing their views, challenges and problems which they might need us to know and improve on them.” “(…) we have suggestion box in our clinic but very few patients use them as most of the elderly people are unable to read or write.”*

(*FGD: Nurses*).

### Utilization of community health workers

Our findings revealed that organization of health services has community health workers known as home based carers to support chronic patients on the compliance and management of their conditions in the community.

#### Quotations

*“(…) home based carers are integrated with our services as they help us by checking or monitoring patients in their households for compliance on medication and also bring the names of patients who might need additional help from the clinic.”*

(*FGD: Nurses*).

*“(…) home based carers help us in the clinic by tracing the patients in the community.” “(…) the home based carers as they are in the community they also assist in referring patients to the clinic and therefore integrating their services with clinic services.”*

(*FGD: Nurses*).

Accounts from the patients indicate that there are patients who do not have trust in home based carers as they perceive them as people who are not educated to provide health services.

#### Quotations

*“(…) the problem is that some of us here we don’t accept Home Based Carers in our households as we think they are not educated.”*

(*FGD: Chronic patients).*

### Support from the traditional authority in health service delivery

There also seemed a minimal involvement from the traditional authority on health service delivery in the study area. The patients would like to see the traditional authority getting involved in mobilizing the community to utilize health services.

#### Quotations

*“(…) our traditional leader should be involved to mobilize people to come to the clinic for testing as the community members respects him.”“(…) even our traditional leader should be able to know what the health problems in the community are and be able to help those who cannot read to know better about preventing diseases.”*

(*FGD: Chronic patients).*

In terms of organization of health services, support groups are organised to support chronic patients on the compliance and management of their conditions in the community. However, not in every clinic patients are willing to engage themselves in these groups. This might be caused by a lack of support from the traditional authority.

#### Quotations

*“(…) the community members and the patients have an opportunity to be part of the support groups we have in the clinic for them to talk about their conditions and link with other patients with similar conditions to form adherence programmes.”*

(*FGD: Nurses*).

*“(…) we only have support groups which some patients are engaged in to discuss health service delivery.” “(…) the participation of the community members sometimes is not that good because when we call them for support groups very few come to attend.”*

(*FGD: Nurses*).

### Need for Knowledge and training in early detection, prevention and control of chronic conditions

Although it is important to ensure that health care professionals and patients are aware of available self-management support, sustainability of these programs and services is founded on patients’ capacity to participate. There is consistent lack of knowledge from patients on the risk factors of chronic diseases and their signs and symptoms. This affects the awareness, prevention and management of chronic diseases.

#### Quotations

“(…) *we need to be taught about the type of food we have to eat in order to control our health conditions”. “(…) I don’t know what caused me to have high blood”. “(…) knowledge is power so we need information in our communities.” “(…) we have to be taught all these here at the clinic but the nurses don’t have time to tell us about these diseases.”*

(*FGD: Chronic patients).*

From the FGDs some patients demonstrated to be able to advise other community members on what to do in order to prevent the onset of chronic diseases. But they also see an important role for the home based carers to distribute health information in the community.

#### Quotations

*“(…) as I discovered that eating lot of salt, sugar and fat caused problems for me, I can advise the people in the community to reduce on those such that they don’t end up like me”.*

(*FGD: Chronic patients).*

*“(…) more information is what we need to manage our conditions…..Yes I agree if we can get health education even in the community we will be able to manage our health problems.” “(…) if the home based carers can be given more information also about the management of these conditions it will help us not to frequently come to the clinic.”*

(*FGD: Chronic patients).*

Despite nurses' role and skills development stimulating a shift away from the acute care model, we observed that nurses had lack of training in chronic disease management. The nurses attended more trainings on HIV, TB, Child Health but little on diabetes, hypertension, mental health or cardiovascular diseases.

#### Quotations

*“(…) with regard to chronic disease management there has never been a training or workshop which I have attended”. “(…) I had never attended any training in relation to chronic disease management”. “(…)I would like to tell them they should consider to train the people who are involved in patient care not only the professional nurses but even the subordinates should be trained. I would like to see them financing the trainings and us having proper equipment’s in the clinic including the cars to visit the patients on the community”.*

(*FGD: Nurses*).

#### Quotations

*“(…) talking for myself, from 2011 I never attended any training or workshop in relation to chronic disease management’… “(…) the same to all of us, we never attended any training or workshop on chronic disease management since the year 2010”. “(…) we only attend trainings on Human Immunodeficiency Virus (HIV) and Tuberculosis (TB)”. “(…) me too I never attended any training in relation to chronic disease management except the HIV trainings”… “(…) most of the trainings are on HIV Counseling and Testing (HCT) and Prevention of Mother to Child Transmission (PMTCT)”.*

(*FGD: Nurses*).

Both chronic disease patients and health care providers have clear preferences and expectations on the needs to improve the care of patients with chronic illness. This covers a range of suggestions from the modalities to receive information, to what to be receive from either the clinics or from health care workers till how to support health services delivery.

#### Quotations

*(…) the right way for us to get information will be to distribute pamphlets with how to manage these diseases.” (…) I think if they can have time to explain to us because most of us we are unable to read this will help us to understand these diseases better and this should be done frequently.” (…) we also want to hear about health information at the traditional authority meetings. If nurses can request to visit the traditional authority it will help those who don’t want to come to the clinic to understand health issues.”*

(*FGD: Chronic patients).*

*“(…) the nurses will be best suitable to give us information about how to manage our conditions because they are trained to do that.”*

(*FGD: Chronic patients).*

*“(…) I would to like hear a lot about these diseases on the radio because it is very rare for us to hear nurses or doctors talking about these diseases on the radio.”*

(*FGD: Chronic patients).*

### Need for more resources, intersectoral collaboration and support from district and or provincial level for chronic disease management

From the FGDs of chronic patients and nurses, it was found that resources for chronic disease management are lacking including shortage of medications and nurses. Most of the drugs were not available at health center level. Thus, patients were referred to hospital where their medications weren’t available either. This forced patients to use out-of-pocket. Though this is beyond the means of many poor households and leads to defaulters to treatment and relapses.

#### Quotations

*“(…) if government can give health department enough money to buy medicines this will improve our lives as we will be having treatment every time we come to the clinic.” “(…) I would like to see the situation of availability of medication improving in our clinic. I agree with her because we are now depending on these medications so they should make sure that medications are always available to save our lives.” (…) I would like to see delivery of medications improving as sometimes we spend months without having some of our medications and this might complicate our health conditions.”*

(*FGD: Chronic patients).*

#### Quotations

*“(…) I think the problem lies with shortage of treatment because chronic diseases needs proper medical maintenance with frequent supply of medication. We sometimes have patients who relapse due to shortage of medications to keep their conditions well and some medications get out of stock for a long time. I can mention metfornin was out of stock for a very long time and now we have a problem with adalat which is out of stock”*

*(FGD: Nurses).*

We found community programmes efforts to support the health system in managing chronic illness and addressing the social determinants of health for prevention.

#### Quotations

*“(…) we have programmes in place like school health services and the home based carers do provide health education to the communities.” “(…) there is Tiangmaatla Home Based Care and Foundation for Professional Development (FPD) which are involved in health activities in the community. The drop in centre assists in taking care of the orphanage children in the community.”*

(*FGD: Nurses*).

*“(…) the programmes are structured well like the door to door campaigns are very effective.” “(…) as the health care workers and the home based carers we are conducting door to door campaigns every three months.” “(…) we also have awareness campaigns frequently to educate the community members about health issues.” “(…) again we visit the local traditional leaders to give them information which they need to disseminate to the community members during funerals.”*

(*FGD: Nurses*).

The nurses have a request to the Department of Health to improve availability of recreational facilities in the study area.

#### Quotations

*“(…) I will raise an issue of the non-availability of physical activity or recreational facilities which we and the chronic disease patients can use to implement healthy lifestyle activities.” “(…) I would like to see government creating facilities and programmes for old age, youth, chronic diseases and even us health care workers we need some recreation facilities to go and refresh our minds.”*

(*FGD: Nurses*).

Chronic disease patients were concerned about the infrastructures in the clinics and nurses had concerns about the shortage of personnel.

#### Quotations

*“(…) me I’m not happy about the waiting area because it’s just an open place and its cold like we are in a fridge.” “(…) we stay outside without chairs nor shelter and is very cold in the morning therefore we are not treated well in that regard.” “(…) I wish the government can improve our clinic and make it bigger as it is very small and sometimes we have to sit outside even in winter.”*

(*FGD: Chronic patients).*

*“(…) we are short staffed in our clinic and these cause patients to wait for a long time in the queues.” “(…) we have many chronic patients in our community so when they come to the clinic we need more health care workers to assist with the work load. You find that most of the patients spend almost the whole day in the clinic which is not good for chronic patients.”*

(*FGD: Nurses).*

## Discussion

Chronic conditions are an increasing burden for health care systems worldwide [1,2] and this has resulted in self-care initiatives and a shift in responsibility to the people living with chronic diseases [21]. The classic health care system focuses much on the provision of acute healthcare as it has been designed to identify and treat individuals and discharge them back to the community. However, the associated risk factors for chronic diseases coupled with health care needs and social circumstances of people living with chronic diseases over a lifetime make the management of chronic conditions more complex [8]. The current study explored the experiences, challenges, barriers to and facilitators for chronic disease management from the patients’ and health care workers perspective by using FGDs. Despite the different conditions of chronic patients and different positions of nurses in the healthcare system, all mentioned similar challenges with regard to lack of knowledge, shortage of medication and shortage of nurses in the clinics. This causes long waiting times for patients in a clinic. Health care workers report they lack knowledge and are poorly trained on the management of chronic diseases. Participants link this with a lack of supervision by the district and provincial health managers together with poor dissemination of guidelines. Both patients and nurses mentioned the need to communicate with traditional healers and integrate their services in order to early detect and manage chronic diseases in the community better.

Our study findings are in accordance with other research findings in many countries where knowledge of the risk factors of chronic conditions and how to manage them is poor [21]. The statement that knowledge was poor among health workers corresponds with findings from a study by de-Graft et. al 2010. Poor knowledge of chronic diseases leads patients and their carers to attribute these diseases to witchcraft. It also initiates problematic treatment practices such as healer shopping within traditional healing systems [22]. Our study findings add to the research knowledge on the interest that both nurses and patients have in improving their collaboration with traditional health practitioners (THPs) to address the burden of chronic diseases in this rural area.

From existing evidence, we know that encounters between health care professionals and chronically ill patients may be variously sporadic or on-going, occasional or intensive, and may involve one primary care provider or an array of specialist practitioners [9]. Our study supports prior research documenting that chronic disease patients have a first contact with HCPs at PHC level [23]. These consultations provide the opportunity for information exchange and successful self-management practices to begin. However, our study documented that in real practice PHC professionals often do not have the resources such as quality equipment’s and promotional materials to assist local community self-management support services such as education programs. Another important barrier for CDM in practice is the lack of continuous availability of medicines plays an essential part in the provision of health care for chronic conditions [24].

The statement that knowledge was poor among health workers corresponds with findings from a study by de-Graft et.al 2010. Poor knowledge of chronic diseases leads patients and their carers to attribute these diseases to witchcraft. It also initiates problematic treatment practices such as healer shopping within traditional healing systems [22]. Our study clearly shows that HCP in the Dikgale area are insufficiently trained both to educate the community on risk factors for chronic conditions as for managing them. This is in line with prior research [22].

Participants mentioned a lack of supervision of health care providers. This is an important issue to address since supervision is a process of helping health staff improve their work performance. All health facilities are supposed to receive supervision from the higher levels and maintain linkages with communities through Community Home Based Carers. Lack of supervision and poor dissemination of guidelines has been found to be a contributing factor to lack of knowledge by health care worker in the clinics. Mayosi et.al, 2009, already stated that the main barrier in the implementation of the guidelines was the insufficient dissemination of the national guideline for the management and control of non-communicable diseases combined with the lack of monitoring and assessment [25].

Improving the health of people with chronic illness requires transforming a system that is essentially reactive - responding mainly when a person is sick - to one that is proactive and focused on keeping a person as healthy as possible [26]. This requires not only determining what care is needed, but defining the roles and tasks for ensuring the patient gets care using structured, planned interactions with different health care providers. It also requires making follow-up a part of standard procedure, so patients are not left on their own once they leave the health care facilities [27,28]. Our study shows that the latter is not currently happening in the study area. Our results also illustrate that patients would like to receive more information on the management of their conditions and to prevent complications.

The study findings also indicated that there is poor referral of patients from traditional healers to the clinics and there is no integration of services. This concurs with findings from the study by Mngqundaniso and Peltzer in South Africa [29] which found that nurses expressed a low regard for traditional healing and nurses practiced low rates of referrals to traditional healers. The referrals from traditional healers were done but mainly in the patient’s interest and not as a last resort for chronic or terminal illness [28]. Although there are some concerns, the potential of involving traditional healers in chronic disease management deserves further consideration.

In summary, this study clearly shows that gaps exist between effective interventions in research studies and what clinicians do in practice, and between what clinicians in their offices recommend to patients and what patients do at home and in their communities. Results detail these gaps and provide handles to develop CDM interventions in the region. These interventions should be developed in such a way that they are oriented towards health promotion and prevention through a primary health care approach in order to effectively respond to the complex social, cultural and behavioural issues associated with NCDs. Therefore, further research will be conducted in the study area to evaluate the planned community intervention programme which is an important component of the strategy to help solve prevention of NCDs [30]. This will include a public health approach which is greatly shaped by community engagement on a variety of levels [31,32] and thus bringing better relationships between community members and social groups, community health workers (CHWs), THPs and public health professionals which are core to health promotion and prevention. One interesting finding of our study is the interest of nurses and patients in improving the collaboration between THP and public health professionals. Exploring knowledge and views of THP on chronic conditions and their role in this is another interesting path for further research.

### Strengths of the study

The readiness of the health care system in rural South African areas to address chronic conditions had not been explored yet so this study filled a gap. The study results are being used to develop an intervention in the region to improve CDM so potentially contributing to the health of the people in the study area.

The interview guides for chronic patients were translated into local language (Sepedi) from English in order for the participants to clearly understand the meaning of the questions. The principal investigator (EM) coded all transcripts and at least one FGD of each group was coded independently by a second researcher (HB or JF) to support validity. Regular discussions (EM, HB, JF) on the emerging themes and codes were held and documented to support reliability.

### Weaknesses of the study

Although we set out to recruit a purposeful sample, most patients who participated were female, potentially missing some of the specific attitudes and experiences of male chronic patients. This might be due to the fact that more males were working during the conduct of the study. Our study findings need to be interpreted cautiously as the Dikgale HDSS is developed as a sub district level surveillance system in a rural setting of Capricorn District of Limpopo Province in South Africa. Therefore, transferability to a larger population at Provincial or country level might be limited. However, findings in our study are in line with most of the findings from other sub-national or national surveys from other countries with a comparable health care system [33].

## Conclusions

There is a need to fully integrate non-communicable diseases into the re-engineering of Primary Health care in South Africa with the view to increasing community based prevention, screening, self-management, care (including rehabilitation and palliative care) and referral according to the WHO innovative model for chronic care [34]. As was seen in this study, there is currently insufficient provision of effective NCD services in primary health care at the Dikgale HDSS. The NCD and health services provided by the three health facilities mainly focused on diagnoses and treatments of diseases, whereas prevention and health promotion were not sufficiently offered and delivered to the target populations. This undermines the development of effective and sustainable primary and secondary care interventions.

Therefore, the study findings will be used to tackle the current challenges, develop and implement interventions in a form of integrated chronic disease management model in primary health care and research to further improve chronic disease management.

## Abbreviations

FPD: Foundation for Professional Development
HCP: Health care provider
HCT: HIV counseling and testing
HDSS: Dikgale health and demographic surveillance site
HIV: Human immunodeficiency virus
NCD: Non-communicable disease
PMTCT: Prevention of mother to child transmission
TB: Tuberculosis
